# Radiation Therapy Personalization in Cancer Treatment: Strategies and Perspectives

**DOI:** 10.3390/ijms26136375

**Published:** 2025-07-02

**Authors:** Marco Calvaruso, Gaia Pucci, Cristiana Alberghina, Luigi Minafra

**Affiliations:** Institute of Bioimaging and Complex Biological Systems—National Research Council (IBSBC—CNR), 90015 Cefalù, PA, Italy; gaiapucci@cnr.it (G.P.); cristianaalberghina@cnr.it (C.A.); luigi.minafra@cnr.it (L.M.)

**Keywords:** radiobiology, radiotherapy, personalized medicine

## Abstract

Modern oncology increasingly relies on personalized strategies that aim to customize medical interventions, using both tumor biology and clinical features to enhance efficacy and minimize adverse effects. In recent years, precision medicine has been implemented as part of systemic therapies; however, its integration into radiation therapy (RT) is still a work in progress. Conventional RT treatment plans are based on the Linear Quadratic (LQ) model and utilize standardized alpha and beta ratios (α/β), which ignore the high variability in terms of treatment response between and within patients. Recent advances in radiobiology, as well as general medical technologies, have also driven a shift toward more tailored approaches, including in RT. This review provides an overview of current knowledge and future perspectives for the personalization of RT, highlighting the role of tumor and patient-specific biomarkers, advanced imaging techniques, and novel therapeutic approaches. As an alternative to conventional RT modalities, hadron therapy and Flash RT are discussed as innovative approaches with the potential to improve tumor targeting while sparing normal tissues. Furthermore, the synergistic combination of RT with immunotherapy is discussed as a potential strategy to support antitumor immune responses and overcome resistance. By integrating biological insights, technological innovation, and clinical expertise, personalized radiation therapy may significantly advance the precision oncology paradigm.

## 1. Introduction

In recent years, personalized medicine has become a pivotal strategy to enhance therapeutic results and patient care in oncology. “Tailored therapies” aim at improving the efficacy of medical approaches based on both biological features and patients’ unique features, thereby minimizing collateral effects and ensuring more precise care. On this ground, the concept of personalized medicine has also been extended to RT to achieve the same goals as those already reached with chemotherapy [1].

Historically, conventional RT has been based on standardized protocols obtained by the application of the LQ model. The LQ model is one of the most used models for estimating the effects of different radiation doses on cell survival after RT and to calculate the tumor control probability (TCP), which represents the probability of completely eradicating all neoplastic cells in a tumor. The α and β parameters and the resulting α/β ratio are the basis of the LQ model, representing, respectively, the unrepairable and repairable radiation-induced DNA damage. To date, RT treatment plans have relied on fixed values of the α/β ratio obtained by averaging the α/β ratios for a specific organ tumor. Consequently, the overall dose delivered is the same for patients with the same organ tumor, despite a strong heterogeneity among tumor subtypes [2]. Hence, the concept of one-size-fits-all, often misused in RT, should be replaced by a more comprehensive understanding of tumor biology and genetic and molecular features of patients, as well as other individual factors useful for customizing specific treatment plans for each patient.

The development of advanced imaging technologies and the identification of cancer biomarkers are of paramount importance in developing personalized RT strategies. Imaging techniques allow us to finely contour the volume of cancer targets, thus treating the tumor bulk with higher precision while sparing the surrounding healthy organs. On the other hand, the identification of specific biomarkers expressed by a tumor may help predict a cancer’s response to ionizing radiations (IR). Moreover, the adoption of new RT approaches relying on the use of hadrons (such as protons and carbon ions) results in a more effective strategy for eradicating radioresistant tumors. In fact, for their physics characteristics, charged particles demonstrate higher ballistic precision and dose distribution compared to X-rays and photons used in conventional RT, thus reducing damage to healthy tissues [3].

In this context, a cutting-edge field of radiobiology research is represented by Flash RT. Flash RT is an innovative technique in the field of radiation oncology. It delivers ultra-high doses of radiation in extremely short time intervals, typically within milliseconds. This approach is currently under investigation for its potential to maximize the therapeutic effect on tumors while minimizing damage to normal tissues [4].

The improvement of personalized therapies is not only limited to the implementation of technologies but also requires a multidisciplinary approach able to integrate different competencies coming from radiobiologists, medical physicists, and radiotherapists. In this context, immunology also represents a branch of research that is synergically linked to RT. The combination of RT with immunotherapies, such as immune checkpoint inhibitors (ICIs), may improve the efficacy of both treatments by modulating the tumor microenvironment (TME) and increasing the immune response [5]. Biomedicine research is currently based on the identification of novel biomarkers, the development of new imaging modalities, and the improvement of treatments that can further increase the efficacy of clinical protocols. Moreover, advances in genomic profiling may drive treatment decisions toward increasingly effective precision oncology.

This review aims to analyze the most recent advances in the strategies that are being adopted in the era of precision medicine to pave the way for more personalized RT.

**Figure 1:**
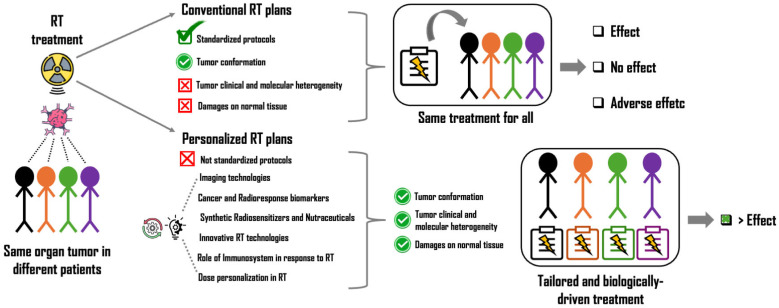
Comparison between conventional and personalized RT plans. Although conventional RT follows standardized protocols (external beam RTusing X or γ-rays), its effectiveness varies among patients. Today, the need for personalized therapies is extremely high, and a multidisciplinary strategy is required for more effective, tailored, and biologically driven RT treatments.

Figure 1 summarizes the main topics that will be discussed in this review.

## 2. Radioresponse Biomarkers

By definition, a biomarker is a “biological indicator” that can include genetic, epigenetic, proteomic, and metabolic markers that are linked to a specific physiological state rather than a pathological condition. In oncology and clinical practice, the identification of cancer biomarkers (CB) is pivotal to predicting prognosis and defining the best care plan for patients. Biomarkers represent the starting point to tailor cancer treatment and can provide insights into the mechanisms of radioresistance, guiding the development of combination therapies that enhance the sensitivity of tumors to radiation. CB can be identified in “in situ” or biological fluids. We refer to “in situ” when their expression is within the tissue where the cancer is located, and they can be represented by specific proteins/enzymes, genetic/epigenetic changes, or histological markers. On the other hand, they can be detected in liquid biopsies like blood, urine, or saliva. Circulating tumor cells (CTCs), circulating tumor DNA (ctDNA), exosomes and microvesicles, circulating proteins, and metabolites belong to this class of bioindicators. Their detection is not an easygoing process and requires resorting to several techniques such as Immunohistochemistry (IHC), Polymerase Chain Reaction (PCR), Next Generation Sequencing (NGS), Flow Cytometry, and Immune assays such as ELISA [6]. For breast cancer (BC), a new approach utilizing Liquid Chromatography-Mass Spectrometry (LC-MS) has been developed to predict response to RT [7].

To develop more personalized radiation oncology, it is important to correlate tumor biomarker expression with RT response. Indeed, there is a direct and strong dependence between tumor intrinsic features and treatment outcome. The effectiveness of RT can depend on various biological factors such as DNA-repair systems, cell cycle checkpoints, intra-tumoral levels of oxygen, the activity of immune system cells (both circulating and resident), and the balance between pro-survival and pro-apoptotic signals. When referring to DNA repair and related genetic markers, certain mutations in key genes like *TP53*, *ATM*, *BRCA1/2*, and *ATR* have been linked to tumor radiosensitivity and/or radioresistance in different types of cancer [8,9]. For example, tumors with non-functional homologous recombination (HR) are often characterized by increased radiosensitivity and may be treated in combination with PARP inhibitors to maximize treatment effects [10].

Genomic modifications such as methylation of the promoters of genes involved in DNA repair (e.g., *MGMT*, *MLH1*) represent predictive biomarkers that can be correlated with RT response [11,12].

Recently, two models have also been developed to predict and quantify tumor intrinsic radiosensitivity based on specific gene expression signatures, the “Radiosensitivity Index” (RSI) and the “Genomic-Adjusted Radiation Dose” (GARD). RSI is an index based on a mathematical algorithm that combines the expression of 10 genes involved in DNA repair, cell cycle control, apoptosis, cell proliferation, hypoxia, and immune response to predict tumor radiosensitivity [13]. GARD integrates the specific expression of several genes in tumor tissues with the physical parameter of RT to personalize IR and to design biology-driven treatments [14]. Both models have been validated in multiple cohorts of patients, showing promising results in terms of the prediction of RT effects.

As regards gene expression signatures, we conducted several in vitro and in vivo studies on different tumor models of BC and glioblastoma and identified radioresponse gene signatures specific both for the tumor type and RT beam delivered, such as X-rays, electrons, or protons. In BC primary cells, we described gene expression signatures activated by different high doses of electron beam (9 Gy and 23 Gy). In particular, we selected a 4-gene signature represented by the genes *AREG*, *FOSB*, *FOS*, and *RRAD* and a 6-gene signature, which also included *GDF15*, *GRIN2C*, and *TAF7L* genes associated with high levels of radioresistance. As regards the roles of these genes, *AREG* promotes tumor growth and radioresistance via EGFR signaling. FOS and FOSB are stress-responsive transcription factors (AP-1 complex) involved in radiation-induced survival and apoptosis pathways. *RRAD* regulates glucose metabolism and can enhance radioresistance by limiting oxidative stress. *GDF15* is a stress-induced cytokine linked to tumor progression, immune evasion, and poor response to therapy. *GRIN2C*, though primarily neuronal, may influence plasticity and resistance in brain tumors. *TAF7L* is involved in transcriptional regulation and may affect cancer cell differentiation and response to radiation [15]. We identified other interesting novel gene signatures in “in vivo” models of BC treated with proton beams and in “in vitro” models of glioblastoma irradiated both with protons and X-rays, also in combination with a targeted c-SRC molecule [16,17,18].

Tumor hypoxia represents one of the main physiological conditions responsible for radioresistance because the low level of oxygen inhibits the fixation of DNA damage and thus protects tissues from IR effects. The combination of Positron Emission Tomography (PET) using hypoxia tracers like [F]18-fluoromisonidazole (18F-FMISO) together with the analysis of the expression of genes involved in hypoxia like *HIF-1α*, *CAIX*, and *GLUT1* may give a deeper insight into hypoxic tumors acquired radioresistance [19]. In addition to the tumor bulk, radioresistance is also linked to the TME and the surrounding cells. Cells of the immune system, including tumor-infiltrating lymphocytes (TILs), PD-L1 expression, and cytokine profiles, are relevant to predicting the efficacy of RT. TIL intratumoral density is indeed a good prognostic marker associated with improved response to RT and patients’ survival outcomes [20]. In contrast, TME-enriched regulatory T cells (Tregs), M2 macrophages, or myeloid-derived suppressor cells (MDSCs) may hamper the efficiency of both RT and immune checkpoint inhibition, suggesting that the presence of immune cells within the tumor or TME-infiltrating immune cells may be considered as a biomarker to effectively stratify patients undergoing RT [21,22].

We studied hypoxia transcriptomic modifications following proton irradiation in the U87 glioblastoma multiforme (GBM) cell line and described gene signatures linked to hypoxia and proton therapy. We also identified some deregulated pathways responsible for the cell death/survival balance involved in GBM radioresistance and potentially useful as molecular targets for the development of new biological drugs [23].

In order to overcome GBM radioresistance, we carried out gene knockdown of key factors involved in the molecular response to hypoxia in the U87 cell line. In particular, *GLUT-3* gene silencing is an effective and promising option for controlling the anaerobic use of pyruvate and better proliferation rate reduction [24].

Together with tissue biomarkers, liquid biopsies allow for monitoring radioresponse, with the advantage of being less invasive. Recent studies have highlighted that ctDNA levels may change after treatment and that its concentration is highly prognostic [25]. Furthermore, specific ctDNA mutational patterns or methylation profiles may predict treatment resistance in solid tumors [26].

In addition, exosomes are gaining attention as markers of tumor radiosensitivity. Exosomes may transport oncogenic microRNAs (miRNAs), proteins, and DNA fragments, which reflect the state of tumor progression. The content of exosomes and their alteration influence intercellular communication and may change over time. Such modifications may provide highly valuable information for the response to therapy [27]. For example, miR-21 and miR-210, usually carried within exosomes, have been associated with the regulation of cancer cell survival and proliferation and, thus, can be considered useful markers for follow-up radiation treatments [28,29].

Proteomic and Mass Spectrometry techniques, such as LC-MS or antibody arrays, are also useful to detect other novel biomarkers that are modulated by IR and linked to clinical outcomes. LC-MS conducted in different types of neoplasms allowed for the identification of several potential biomarkers of radioresponse and recurrence. As an example, serum levels of ADIPOQ, HEY2, and FUT10 proteins were highlighted as potential predictive biomarkers for the treatment response of head and neck cancers [30].

Metabolomic techniques with the identification of changes in metabolites are another promising strategy from the perspective of personalized RT. Molecules involved in glycolysis, oxidative phosphorylation, and lipid metabolism may serve as early indicators of radiation-induced stress and adaptive responses [31].

For instance, increased lactate production has been observed in resistant glioblastoma cells, suggesting a metabolic rewiring that supports survival and radioresistance [32].

Despite all these advancements, it is still challenging to introduce biomarkers as targets for anti-tumor therapies. Cancer heterogeneity and technical variability in biomarkers detection and quantification still represent a limitation. Validation in large prospective cohorts of cases and standardization of assay platforms are essential to establish clinical utility and reproducibility.

## 3. Mechanisms of Cancer Cell Radioresistance

The success of RT in cancer treatment relies on the ability to overcome tumor radioresistance. Radioresistance can be classified as intrinsic and extrinsic. Intrinsic radioresistance is linked to genetic and epigenetic features, which can improve tumor DNA repair capacity, lead to defects in apoptosis, and are linked to the presence of cancer stem cells. Extrinsic radioresistance is instead related to TME-dependent factors, which encompass hypoxia, altered vasculature, immune evasion, and stromal interactions [33]. Several factors enable cancer cells to survive and proliferate despite the genotoxic stress induced by IR, which is mainly induced by the formation of DNA breaks, particularly DNA double-strand breaks (DSBs). DSBs are the most lethal form of lesions caused by IR, and they trigger two different molecular pathways aimed at their repair: the homologous recombination (HR) and non-homologous end joining (NHEJ) [34]. The upregulation of proteins involved in both mechanisms represents a strategy adopted by cancer cells to resist IR-induced DNA damage. For instance, the overexpression of ATM, DNA-PKcs, or RAD 51 (master regulators of HR and NHEJ) is associated with radioresistance in cancer cells, and the use of their inhibitors could restore the efficacy of RT [35]. Also, the dysregulation of the cell cycle enables tumor cells to bypass cell-cycle stops and allows them to keep on proliferating despite the formation of DNA breaks. Cell cycle arrest can promote radioresistance in cancer cells by providing time for DNA repair mechanisms to function, potentially mitigating the effects of radiation-induced DNA damage [36]. For example, increased activation of checkpoint proteins such as CHK1 and WEE1, which mediate G2/M arrest, is linked to cancer radioresistance, and inhibitors have been proposed to enhance radiosensitivity [37]. Also, the resistance to programmed cell-death mechanisms (apoptosis) plays a pivotal role in tumor radioresistance; the over-expression of anti-apoptotic proteins like BCL-2 and the concurrent decrease of pro-apoptotic signals like BAX, alters the apoptotic balance and counteracts the effects of IR on tumor cells [38]. Another crucial mechanism leading to radioresistance is tumor hypoxia. As previously mentioned, the reduction of pO_2_ within the tumor bulk is responsible for the reduction of reactive oxygen species (ROS) formation. ROS are key players in the indirect damage induced by IR at the cellular level; hence, a diminished ROS production contributes to RT treatment failure [39]. Furthermore, hypoxia induces the activation of hypoxia-inducible factors (HIFs), which, in turn, stimulate angiogenesis and cell proliferation, thus supporting radioresistance [40]. Finally, cancer stem cells (CSCs) are closely associated with radioresistance because of their highly efficient DNA repair ability, reduced ROS levels, and the activation of pro-survival pathways, including WNT, NOTCH, and PI3K/AKT. The hypoxic tumor niches in which they reside additionally shield them from radiation insult. These features allow CSCs to escape from treatment and promote tumor recurrence. CSC-specific targeting could provide a novel treatment strategy to reverse radioresistance and RT effect [41].

Recent evidence suggests that long-non-coding RNA (LncRNAs) are involved in cancer radioresistance. LncRNAs are a class of RNA molecules longer than 200 nucleotides that do not code for proteins but are still able to regulate gene expression and several cellular processes, including the activity of miRNAs (another class of noncoding RNA with regulatory functions) [42,43]. In the context of cancer RT, the expression of different lncRNAs is modulated; hence, the identification of those that are inhibited or activated by IR might be useful to enhance the sensitivity of cancer cells to RT [44]. In radioresistant BC, for instance, the lncRNA DUXAP8 is overexpressed and its expression is correlated to poor prognosis. DUXAP8 is involved in the regulation of cell growth, proliferation, and survival by modulating the PI3K/AKT/mTOR pathway. Targeting DUXAP8 may be useful as a therapeutic strategy for improving RT efficacy in radioresistant BC tumors [45]. In gastric cancers, lncRNA DNAH17-AS1 induces cancer proliferation and radioresistance by competing with the tumor suppressor miRNA miR-202-3p [46]. However, in gastric cancer, many other lncRNAs have been identified that are involved in cancer radioresistance by promoting DNA damage repair mechanisms, cell cycle arrest, and inhibition of apoptosis [47]. The LncRNA HOTAIR is involved in radioresistance in colon-rectal cancer by interacting with ataxia-telangiectasia mutated- and Rad3-related (ATR) and promoting DNA damage response [48]. In Nasopharyngeal Carcinoma, the lncRNA CASC19 is involved in radioresistance by promoting cancer cell autophagy. This effect is given by the interaction with miR-340-3p, a tumor suppressor miRNA that targets genes related to proliferation, apoptosis, and metastasis [49,50].

The regulation of miRNAs by lncRNA underlines how both classes of noncoding RNA are of pivotal importance in regulating the effects of IR in cancer. miRNAs are smaller than lncRNA (not longer than 22 nucleotides), and lncRNA acts as a sponge on the first, competing for the regulation of miRNA target genes [51]. As for lncRNA, miRNAs are also correlated with cancer aggressiveness, and several of them have been identified as responsible for cancer radioresistance [52]. For example, miR-214 is responsible for the degradation of the tumor suppressor PTEN and PI3K/AKT activation, thus inducing radioresistance of ovarian cancer cells [53]. In colorectal cancer, miR-183-5p decreases the mRNA of ATG5, an autophagy-related protein that interacts with PI3K and WNT pathways [54]. On the contrary, miR-4537 and miR-365 act as tumor suppressors and enhance radiosensitivity in gastric and non-small cell lung cancers, respectively [55,56].

MiR-21, a frequently associated miRNA in cancer, shows consistent upregulation in multiple neoplasms, including breast cancer and glioblastoma. It can target different molecules involved in cancer-signaling pathways controlling cell survival and apoptosis, such as TP53, PI3K/AKT, and RAS, which are significant targets for increasing radiosensitivity [57]. Therefore, miRNAs as well as LncRNAs, beyond those mentioned, represent promising biomarkers and therapeutic targets with the potential to overcome radioresistance and improve the cancer patient prognosis.

**Figure 2:**
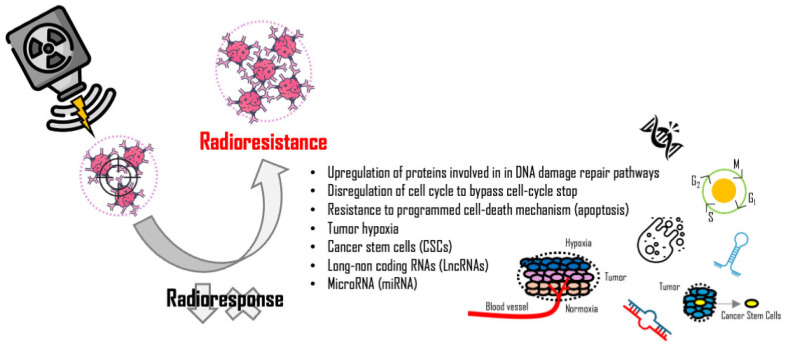
Main mechanisms of cancer radioresistance.

All these mechanisms underline the complexity of tumor radioresistance, characterized by an intricate network of factors, including proteins and gene expression regulators (noncoding RNAs), which affect the efficacy of RT (Figure 2). Combined strategies exploiting the synergistic effects of RT in combination with radiosensitizer administration could lead to more effective therapeutic approaches.

## 4. Synthetic Radiosensitizers and Nutraceuticals

Although RT still represents one of the three pillars on which cancer treatment stands, its efficacy is often hampered by the intrinsic or acquired radioresistance of tumor cells. Tumor radioresistance requires the development of strategies to enhance tumor radiosensitivity without affecting normal tissue integrity [3]. In this context, radiosensitizers are a group of molecules that increase the effects of IR and may be used as an adjuvant in clinical practice and may synergize with RT. Radiosensitizers can be classified into synthetic and non-synthetic agents; the latter are also referred to as nutraceuticals. The goal of both classes is to improve the therapeutic index of RT by potentiating tumor cell kill while minimizing collateral damage to surrounding healthy tissue.

Synthetic radiosensitizers are designed to interfere with targeted molecules and pathways, mainly involved in DNA damage repair, cell cycle regulation, and restoring intratumoral levels of oxygen. Tumor hypoxia is, as reported above, one of the factors that are mainly involved in tumor-acquired radioresistance. Halogenated pyrimidines like 5-bromo-2′-deoxyuridine (BrdU), hypoxic cell sensitizers such as nitroimidazoles, and inhibitors of DNA repair proteins (such as PARP inhibitors) are examples of synthetic radiosensitizers [58,59,60]. Since the anticancer effects of RT are mainly caused by the generation of breaks to the DNA helix, these compounds counteract the cell’s ability to repair sub-lethal DNA damage, promoting apoptosis, or mimicking oxygen to overcome hypoxia-induced radioresistance. However, the introduction of synthetic radiosensitizers is sometimes challenging due to their systemic cytotoxicity, which limits their application. To overcome such limitations, a growing interest has been focused on natural compounds, nutraceuticals, which can exert cell radiosensitization without causing detrimental cytotoxicity.

The clinical relevance of the abovementioned classes of radiosensitizers has been explored in both translational research and clinical oncology. Among synthetic radiosensitizers, 5-fluorouracil (5-FU) remains one of the most characterized. Moreover, 5-FU has been shown to enhance radiosensitivity by inhibiting DNA synthesis and repair pathways in different types of cancer, in glioblastoma and colon cancer cell lines [61], and also “in vivo”. Large, randomized trials such as the German “CAO/ARO/AIO-94 study” have demonstrated a significant improvement in preoperative chemo-radiotherapy when administered concurrently with 5-FU [62]. Similarly, cisplatin (a platinum-based chemotherapeutic) has been shown to induce radiosensitization when administered together with RT. The combination of cisplatin and RT represents, in fact, a common strategy for treating different types of cancers [63]. From the molecular point of view, cisplatin can crosslink with purine bases of the DNA, causing the blocking of DNA and RNA polymerase. Such crosslink eventually induces the failure of DNA damage repair and leads cells to activate apoptosis [64]. Cisplatin’s activity was also exploited to sensitize head and neck and cervical cancers [65,66].

An interesting class of radiosensitizers is represented by hypoxic radiosensitizers, specifically designed to overcome tumor hypoxia. Hypoxia hampers the effectiveness of RT because oxygen is required to fix radiation-induced DNA damage generated by ROS. Historically, nitroimidazoles, such as metronidazole, misonidazole, etanidazole, and nimorazole, were identified as promising hypoxic sensitizer candidates. However, the neurotoxicity of nitroimidazoles limits their use in clinical practice; hence, their clinical application was not further investigated. For this reason, new synthetic compounds such as RRx-001 were proposed and introduced in clinical trials as an adjuvant of RT for the treatment of brain tumors [67].

On the other hand, to restore intratumoral levels of oxygen, hyperbaric oxygen was also used in association with RT and showed an enhanced tumor-radiosensitization [68]. Additionally, another class of radiosensitizers aimed at targeting the protein PARP (e.g., Olaparib and Veliparib) has been proposed as a new therapeutic option, particularly in tumors with defective homologous recombination. These drugs selectively inhibit DNA repair pathways and limit radiation-induced DSB repair. Their use in combination with RT is being intensely explored in clinical trials for a variety of tumors, including glioblastoma, triple-negative BC, and ovarian cancer [69].

Similarly to PARP inhibitors, other small-radiosensitizers have been developed and tested to target the activity of DNA damage response proteins. The selective inhibition of proteins like ATM with the compound AZD0156, or DNA-PKcs with Peposertib (M3814), showed the impairment of DSBs repair and the enhancement of radiation-induced cytotoxicity “in vitro” and “in vivo” [70,71]. Another class of inhibitors is represented by the ones targeting histone deacetylase (HDAC). HDAC inhibitors induce chromatin decondensation, thus exposing DNA to IR. Several compounds (e.g., Vorinostat, Panobinostat, Romidepsin) have been tested in vitro and in vivo models, demonstrating the enhancement of both conventional and particle RT [72].

Inhibitors of WEE1 (a serine/threonine kinase regulating the G2/M checkpoint) like Adavosertib (AZD1775) have been shown to overcome increased G2/M blocks occurring in cancer cells to counteract DNA damage. The encompass of G2/M checkpoints by WEE1 inhibition proved to synergize with RT in human esophageal, cervical, head, and neck cancers [73]. Similarly, CDK4/6 inhibitors like Palbociclib radiosensitize tumors by impairing cell cycle progression and DNA repair capability in vitro and in vivo in several tumor models [74].

Epidermal growth factor receptor (EGFR) inhibitors (e.g., Cetuximab, Erlotinib) have also demonstrated efficacy as radiosensitizers. Inhibition of EGFR impairs DSB repair, causing accumulation of DNA damage after radiation and leading to increased cancer cell death in human non-small cell lung cancer and head and neck squamous cell carcinoma [75,76].

Lastly, the restoration of vascular formation, which is altered during cancer progression, can improve the efficacy of RT. The inhibition of the Vascular Endothelial Growth Factor (VEGF) and its pathway with anti-angiogenic drugs (e.g., Bevacizumab, Sunitinib), promotes vascular normalization and enhances the effects of RT in situ models of glioblastoma, head and neck cancer, lung cancer, melanoma, and colon cancer [77].

Due to their role in mitigating the response to IR, a powerful strategy to improve cancer radiosensitivity is represented by the therapeutic use of lncRNA and miRNA as targets. However, rapid degradation and off-target effects hamper the clinical application of ncRNA-based radiosensitization. For this reason, nanoparticles such as liposomes, exosomes, and dendrimers may be exploited as carriers for ncRNAs, miRNA, or small interfering RNAs (siRNAs) to prevent their fast degradation and restore cancer radiosensitivity by incorporation into cells and releasing their content [78].

Nutraceuticals are mainly derived from dietary sources, and historically, they have been widely used in complementary medicine approaches such as traditional Chinese medicine. These compounds include polyphenols (e.g., curcumin, resveratrol, quercetin), flavonoids (e.g., genistein, apigenin), and other phytochemicals which have been shown to modulate several molecular pathways associated with cell survival, oxidative stress, inflammation, and angiogenesis. Another interesting and positive aspect of nutraceuticals is their dual role: while they can sensitize tumor cells to radiation-induced damage, they can have radioprotective effects on normal tissue. Such balanced activity stems from an overall difference in redox status, metabolic activity, and molecular signaling dynamics between malignant and healthy cells. Hence, due to their generally favorable safety profiles and low toxicity, natural radiosensitizers represent an appealing alternative to synthetic ones, especially for patients with debilitated and highly compromised physical conditions [79].

Nutraceuticals may drive tumor-radiosensitizing effects with a more tolerable approach. Among them, Curcumin, Resveratrol, Genistein, and Quercetin may be referred to as some of the most representative ones that have been investigated for their radiosensitizing effect. Curcumin, a compound derived from Curcuma longa, can modulate numerous signaling pathways, including NFκB and COX-2, reduce oxidative stress, and induce apoptosis in irradiated BC cells. We performed an “omic” study at the transcriptomic and metabolomic level to highlight the action mechanisms of a new formulation of Curcumin, carried with lipid nanoparticles, in producing radiosensitivity effects on the three different BC cell lines [80]. Moreover, Curcumin’s potential is being tested in early-phase clinical trials, where it showed promising results with enhancement of both chemotherapy and radiation efficacy and also a reduction of treatment-related toxicity [81].

Also, Resveratrol, a polyphenolic compound found in grapes and berries, has demonstrated synergistic effects with radiation in cancer models. Its activity relies on supporting ROS accumulation and impairing survival pathways such as PI3K/AKT [82]. Finally, Genistein, an isoflavone from soy, and Quercetin, a flavonoid found in onions and apples, have both been investigated for their radiosensitizing potential in cancer treatment [83,84].

All these compounds interfere with key cellular processes, including DNA repair, heat shock protein expression, and mitochondrial apoptosis, often exerting differential effects between tumor and normal cells [85]. However, because of their low bioavailability and variable composition, nutraceuticals have not been fully integrated into clinical practice yet. Nonetheless, nanotechnology, encapsulation techniques, and targeted delivery systems are under development to reach more effective and consistent clinical applications [86].

## 5. Role of the Immune System in Response to Radiotherapy

Tumor control in RT is primarily achieved by the induction of DNADSBs, which leads to cancer cell death by apoptosis, necrosis, or mitotic catastrophe. However, such effects are not only limited to the tumor bulk but, together with the effects on healthy tissue, RT also affects the immune compartments of the body, which trigger immunomodulatory phenomena followed by the reshaping of the TME and the activation of the anti-tumor immune response [87]. RT may play an immunologic adjuvant role by inducing immunogenic cell death (ICD), a process that contributes to immunogenic modulation by the release of molecules that act as “danger signals”, known as damage-associated molecular patterns (DAMPs). DAMPs include calreticulin exposure on the cell surface, ATP secretion, and high-mobility group box 1 (HMGB1) release into the extracellular compartment, thus promoting an inflammatory response [88].

All these signals lead to the recruitment and maturation of antigen-presenting cells, like dendritic cells (DCs), which can uptake and process tumor antigens to present them to cytotoxic CD8+ T-lymphocytes (CTLs). Hence, RT can drive adaptive immune anti-tumor response by the activation of CTLs. Furthermore, IR can enhance the expression of major histocompatibility complex (MHC) class I molecules on tumor cells and tumor-associated antigens (TAA), which can prime T cells favoring their infiltration within the tumor [89]. On the other hand, the ability of RT to elicit immune-mediated anti-tumor response can also be proved by the occurrence of the “abscopal effect”, where RT treatment has been shown to induce tumor regression also on body districts outside and distant from the irradiated ones [90].

However, RT alone is not capable of producing a persistent anti-tumor effect due to immunosuppressive elements that populate the TME. The TME is populated by regulatory T-cells (Tregs), myeloid-derived suppressor cells (DSCs), and tumor-associated macrophages (TAMs) with an M2 phenotype, that collectively switch off anti-tumor effects by producing immunosuppressive factors such as transforming growth factor-beta (TGF-β) and interleukin-10 (IL-10) [91].

To counteract the activity of the immunosuppressive TME, ICIs which block the inhibitory receptor programmed cell death protein-1 (PD-1) and its ligand (PD-L1) or the cytotoxic T-lymphocyte-associated antigen 4 (CTLA-4), have shown promising results and can restore the anti-tumor effects of T-cell effectors [92]. RT can upregulate PD-L1 expression in both tumor and stromal cells; hence, the combination of RT and ICIs could improve anti-tumor response [93,94].

The strategy to combine RT and ICIs is often referred to as “immune-radiotherapy” (ImmunoRT) and can facilitate the recruitment of effector T cells to the tumor site and reduce immunosuppressive activities, hence improving the overall response to RT [95]. Also, the RT parameters play a key role in influencing immunological activation. The total dose, together with the dose per fraction and the fractionation schedule, influences the immunological outcomes. Hypofractionated regimens, characterized by a small number of fractions in favor of a higher dose per treatment session, have shown an increased efficacy in inducing immunogenic effects compared to conventional low-dose fractionation schedules [96].

Even the timing of ICIs administration with respect to RT can be a crucial factor in terms of immunological or clinical outcomes. Clinical data suggest that the administration of ICIs concurrently with RT, or when using times between treatments lower than 30 days, can have a positive impact on the therapeutic strategy [97].

Despite this encouraging evidence, clinical challenges remain for the implementation of ImmunoRT. The identification of specific biomarkers such as tumor mutational burden (TMB), PD-L1 expression levels, and the profile of circulating immune cells of the patient is an essential feature that may help predict the response to combined treatments [98]. Furthermore, the correlation between immune-related adverse events (irAEs) and immunotherapy should be carefully evaluated when considering submitting patients to the combination of RT and immunotherapy [99]. Given the general and effective advantages of the implementation of RT treatments with immunotherapy, several clinical trials are investigating the effect of ImmunoRT on different types of neoplasms (Table 1). RT induces DSBs and, in turn, leads to the activation of the ATM/ATR signaling, which promotes the upregulation of PD-L1 [94]. The increasing expression of PD-L1 in combination with ICIs has proved to be particularly effective. In fact, recent clinical trials like the phase III PACIFIC demonstrated that PD-L1 blockage with Durvalumab post-chemoradiation significantly improved overall and progression-free survival in unresectable stage III non-small cell lung cancer (NSCLC) [100]. Similarly, the CheckMate-577 trial showed that adjuvant Nivolumab, an anti-PD1, after chemoradiation and surgery, nearly doubled disease-free survival in esophagogastric cancer [101]. Most recently, the ADRIATIC trial revealed that Durvalumab following chemoradiation boosted 5-year overall survival to 55.9 months in limited-stage small cell lung cancer (SCLC), establishing a new standard of care [102]. Additionally, the phase II trial NJLCG 1902 showed that neoadjuvant and adjuvant Pembrolizumab, an anti-PD1, combined with surgery and radiochemotherapy, improved median cancer-free survival in head and neck squamous cell carcinoma patients [103]. These findings reflect a rapidly evolving paradigm in which combination strategies are being optimized to fully exploit the radiosensitizing potential of immunotherapy in cancer treatment.

Future research should better elucidate the mechanism underlying the interplay between RT and ICIs to transform RT from a local treatment modality to a more systemic therapeutic approach to improve long-term tumor control in radioresistant tumors unresponsive to conventional RT.

## 6. Clinical Trials Investigating the Impact of Hypofractionated RT Strategies

In the context of highly radioresistant tumors, a more biologically driven and personalized RT would be auspicable and would lead to the design of treatment schedules based on peculiar and specific biological features of tumors, which are generally heterogeneous and differentially respond to standard RT regimens.

Typically, conventional RT schedules are based on administration of daily fractions of approximately 2 Gy, which can balance the differential repair capabilities of tumor versus normal tissues.

However, in the case of tumors characterized by high repopulation rates, functional DNA damage repair response, and hypoxic conditions, standard regimens may fail to reach an effective tumor control probability [36]. Hypofractionated RT, consisting of delivering larger doses per fraction over a reduced number of treatment sessions, may represent a therapeutic strategy to counteract tumor radioresistance. Indeed, hypofractionation is increasingly being recognized not just as a logistical improvement in cancer care but also as a strategic approach to personalize RT based on tumor biology, patient characteristics, and technological capabilities.

RT treatment plans are based on a mathematical model that puts into relationship the radiation-induced cell killing and DNA damage repair, the so-called LQ model. The LQ model is given by the equation S = e^(−αD − βD2)^, where S is the surviving fraction, D is the dose, and α and β represent the linear and quadratic components of cell killing, respectively [104].

However, due to the high level of heterogeneity among cancers (also among those affecting the same organs) that is caused by the different rates of repopulation and cancer stem cells, the LQ model could be limiting. Different extensions of the LQ model were proposed in recent years, which consider other biological aspects of the tumor with the aim of developing more tailored and biologically driven RT approaches [2]. We recently proposed an implementation of the LQ model, which put into relation “S” with the tumor cell’s doubling time (Td) and the number of clonogens with stem cell features (k), in addition to the α and β parameters, to create a more personalized approach for RT planning. The novelty of our work was to obtain each parameter experimentally from each cell culture tested, while in other published works, these parameters were generally minimized [105].

The ratio between α and β parameters, α/β, is an indicator of tumor sensitivity, and its value reflects cancer radioresistance. Low values of α/β are usually associated with slow proliferation and an efficient capability to repair sublethal damage. Hence, tumors with a low α/β should be more responsive to hypofractionation. Examples of late-responding tumors characterized by low α/β are prostate cancer, which has an overall ratio of 1.5 Gy, breast cancer with an average α/β of 3.5 Gy, melanoma with α/β of 2.5 Gy, and sarcomas with α/β of 4 Gy [106,107,108,109]. In these cases, larger fraction sizes should produce higher biological effects.

On the contrary, early responder tumors with high α/β, such as Head and neck squamous cell carcinoma (HNSCC) with α/β of 10 Gy, Glioblastoma with α/β of 8 Gy, and Lymphomas with α/β of 10 Gy, should be less reliant on dose escalation and hypofractionated regimens [110,111,112].

In clinical trials, hypofractionated RT has shown promise across a variety of tumor types (Table 2). In prostate cancer, many randomized trials have shown that hypofractionation is not inferior to conventional fractionation in terms of biochemical control, with equivalent or even lower toxicity profiles [113]. Long-term follow-up from the UK START trials revealed the safety and efficacy of hypofractionated whole-breast RT in early-stage BC, prompting broad adoption [114]. For the treatment of BC, the FAST FORWARD phase III trial demonstrated the safety of delivering adjuvant RT for early BC 26 Gy in 5 fractions over one week instead of the conventional 40 Gy in a 15-fraction schedule. The study confirmed that ultra-hypofractionation offers comparable tumor control and cosmetic outcomes, with no increase in late toxicity [115]. More recently, the Phase III CHARM trial showed that a shorter course of post-mastectomy radiation plan (16 fractions with a total dose of 42.56 Gy vs. the standard one of 25 fractions with a total dose of 50 Gy), combined with breast reconstruction, was safe and effective [116].

Regarding prostate cancer patients, the phase III trial HYPO-RT-PC demonstrated that ultra-hypofractionated RT (consisting of 42.7 Gy in 7 fractions), although higher acute toxicity, was well tolerated with respect to conventional RT regimens (consisting of 78 Gy in 39 fractions) [117]. Also, the phase III trial PACE-B proved that a total dose of 36.25 Gy in 5 fractions (delivered using SBRT) was non-inferior and with an acceptable acute toxicity profile with respect to the standardized RT regimen for localized prostate cancer [118].

In NSCLC, moderate Hypo-RT has shown promising results by improving the overall survival of patients with stage III NSCLC, while limiting side toxic effects [119].

Overall, hypofractionated RT represents a powerful approach to overcoming cancer radioresistance. It can provide better tumor control probability, and it can be useful to design individualized RT plans. However, the development and improvement of RT-response prediction models and validation of hypofractionated regimens in large-scale clinical trials will be essential to fully include such therapeutic options in future clinical practice.

## 7. Innovative Radiotherapy Technologies

Modern RT is no longer limited to the application of conventional photon or X-ray beams. Conventional RT is based on the delivery of standardized IR fractions, and the principle behind such methodologies has basically not changed throughout the past century. In recent years, to maximize precision toward the tumor target, RT has evolved and now represents a technologically sophisticated field in order to reach a more personalized dose distribution. Hence, RT is going beyond the simple concept of “delivering a dose”, to increase tumor control while minimizing damage to healthy tissue. In this context, new techniques such as stereotactic body radiotherapy (SBRT), volumetric modulated arc therapy (VMAT), hadron therapy, and emerging concepts like FLASH RT and minibeam therapy are paving the way to more tailored approaches.

SBRT represents the most significant evolution in terms of external beam RT. The advantage of SBRT is the delivery of very high doses per fraction in a small number of therapeutic sessions (usually 1–5) with sub-millimeter accuracy. Supported by advanced imaging and patient motion control, SBRT is particularly useful when the cancer target is small and located in organs where the sparing of surrounding healthy structures is pivotal. For this reason, it is applied to treat lung, liver, spine, or prostate cancers [120,121,122,123]. SBRT is also advantageous in treating oligometastatic disease and in medically inoperable patients, offering an alternative to surgery in specific cases [124,125].

VMAT is an advanced form of intensity-modulated radiation therapy (IMRT) that allows the delivery of IR while the linear accelerator (LINAC) rotates around the patient. During rotation, both the beam’s shape and intensity are modulated [126]. VMAT treatments optimize tumor coverage because they conform to the three-dimensional shape of the tumor, thus minimizing irradiation in tumor-surrounding normal structures. Moreover, this technique results in faster treatments with respect to IMRT [127] and it is useful for the treatment of complex tumors such as head and neck and prostate cancers [128,129]. The primary drawbacks of VMAT are the setup costs and the extra time required for the clinical staff to plan the therapy. Additionally, a greater portion of a patient’s body is exposed to low-dose radiation since the total body dose (also known as the integral dose) is higher than that of conformal treatment [130].

Hadrontherapy, also known as particle therapy, is an advanced modality of external beam RT that utilizes charged particles, most commonly protons and carbon ions, instead of traditional X-rays to treat cancer. Due to their physical nature, hadrons deposit the majority of their energy at a specific depth in tissue, more precisely within the tumor, corresponding to the so-called “Bragg peak”. In contrast to photons, which deposit energy over their entire course through the living matter. The advantage of RT-based hadrons is to achieve an unmatched degree of dose precision, which is ideal for pediatric tumors, tumors close to critical organs such as brain tumors (as gliomas), uveal melanoma, and also breast cancers. We conducted several radiobiological studies, both “in vitro” and “in vivo”, on different tumor models of BC, GBM, and also uveal melanoma by using proton beams at the Catana hadrontherapy facility of the LNS-INFN in Catania (Italy). These studies have allowed us to evaluate the cellular and molecular response to hadrontherapy and to identify new radioresponse biomarkers [16,17,131,132].

Hadrontherapy, in particular carbon ion therapy, is moreover effective for highly radioresistant tumors and hypoxic tumors [133]. Although hadrontherapy is currently not widely available due to its high cost and infrastructural requirements, it is at the forefront of technological and radiobiological advancement.

Two promising approaches for preclinical and early-phase clinical research are FLASH-RT and minibeam-RT. FLASH radiation delivers electron or proton beams with ultra-high dose rates (usually more than 40 Gy/s) in extremely short shots (less than a second), resulting in the “FLASH effect”, where tumor control remains effective and normal tissue toxicity is significantly reduced. The main advantage of FLASH-RT is the possibility of treating tumors that are currently not treatable effectively, such as locally diffuse tumors (e.g., brain metastases) and/or radioresistant tumors, located inside or near an organ at risk, which therefore limits the possibility of increasing the dose. The molecular mechanisms underlying the flash effect are still under investigation, but preclinical research on different animal models and human feasibility trials indicate great therapeutic promise [134].

Minibeam-RT is a highly innovative technique based on both X-ray and proton irradiation. Minibeam exploits arrays of very narrow, spatially fractionated beams to create alternating areas of high and low dosage within the tissue. Such spatial conformation of the beams apparently elicits unique biological responses, resulting in tissue sparing and improved tumor selectivity. When paired with FLASH dose rates (as in FLASH minibeam therapy), the synergy has the potential to protect normal tissues during high-dose treatments, particularly in the brain and pediatric applications [135].

To further improve these cutting-edge techniques, the next step would be to integrate them with adaptive planning and biologically guided radiation, in which treatment responds dynamically to changes in tumor biology and the patient’s biological features. Artificial intelligence and machine learning will hopefully help deliver and monitor RT, making it more efficient and tailored in the future [136].

## 8. Conclusions

**Figure 3:**
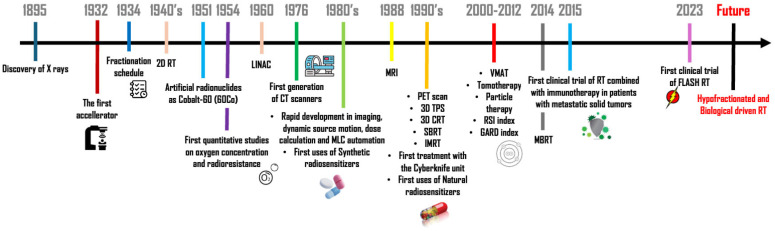
A brief history of RT: from the past to future perspectives. A historical timeline describing all the milestones of RT development, from basic to more personalized approaches. (MRI—Magnetic Resonance Imaging; PET—Positron Emission Tomography; TPS—Treatment Planning System; CRT—Conformal Radiation Therapy; SBRT—Stereotactic Body Radiation Therapy; IMRT—Intensity-Modulated Radiation Therapy; VMAT—Volumetric Modulated Arc Therapy; RSI—Radiosensitivity Index; GARD—Genomic-Adjusted Radiation Dose; MBRT—Minibeam Radiation Therapy).

The personalization of RT would allow us to elevate it to the same level of precision achieved by chemotherapy, which over the years has become progressively more tailored. Such a goal should be coupled to maximize therapeutic efficacy while minimizing toxicity. The identification of radioresponse biomarkers and the administration of radiosensitizers (both synthetic or of natural origin) enable more precise patient stratification and targeted treatments. Innovative RT technologies, such as particle therapy and FLASH-RT, are further improving the degree of treatment accuracy while preserving the integrity of normal tissues. Furthermore, elucidating the interplay between RT and the immune system opens new strategies for combined therapeutic interventions. Finally, hypofractionation could represent a rational approach to tailor treatments based on tumor characteristics. Together, all these strategies allow us to reach more individualized, effective, and patient-centered RT, along with technological implementations. Figure 3 shows the main steps of progress in RT, which, over time, are enabling the scientific community to adopt an increasingly personalized approach.

## Figures and Tables

**Table 1:** Recent clinical trials investigating the promising effect of ImmunoRT in different types of neoplasms.

Immunotherapy and RT Combinations: Clinical Trials				
Clinical Trial	Molecule (ICI)	Pathway Target	Cancer Type	Ref.
PACIFIC Trial	Durvalumab	PD L1 blockage	Non-Small Cell Lung Cancer	[100]
CheckMate 577 Trial	Nivolumab	PD 1 blockage	Esophagogastric Cancer	[101]
ADRIATIC Trial	Durvalumab Durvalumab plus Tremelimumab	PD L1 blockage PD-L1 and CTLA-4 blockage	Small Cell Lung Cancer	[102]
NJLCG 1902 Trial	Pembrolizumab	PD 1 blockage	Head and Neck Squamous Cell Cancer	[103]

**Table 2:** Recent clinical trials investigating the impact of hypofractionated RT strategies across a variety of cancer types.

Hypofractionated RT Strategies: Clinical Trials			
Clinical Trial	RT Plans Specification	Cancer Type	Ref.
UK START Trial	41.6 Gy or 39 Gy in 13 fractions vs. 50 Gy in 25 fractions, over 5 weeks	Breast Cancer	[114]
FAST FORWARD Trial	26 Gy or 27 Gy in 5 fractions (over 1 week) vs. 40 Gy in 15 fractions (over 3 weeks)	Breast Cancer	[115]
CHARM Trial	42.56 Gy in 16 fractions vs. 50 Gy in 25 fractions, 5 days per week	Breast Cancer	[116]
HYPO-RT-PC Trial	42.7 Gy in 7 fractions (3 days per week for 2.5 weeks) vs. 78 Gy in 39 fractions (5 days per week for 8 weeks)	Prostate Cancer	[117]
PACE-B Trial	36.25 Gy in 5 fractions (1 or 2 weeks) vs. 78 Gy in 39 fractions (7.5 weeks) or 62 Gy in 20 fractions (4 weeks)	Prostate Cancer	[118]
A Multicenter, Randomized Phase 3 Trial	60 Gy in 20 fractions vs. 60 Gy in 30 fractions, 5 days per week	Non-Small Cell Lung Cancer	[119]
